# Intraoperative radiotherapy (IORT) combined with external beam radiotherapy (EBRT) for soft-tissue sarcomas – a retrospective evaluation of the Homburg experience in the years 1995–2007

**DOI:** 10.1186/1748-717X-4-32

**Published:** 2009-08-26

**Authors:** Marcus Niewald, Jochen Fleckenstein, Norbert Licht, Caroline Bleuzen, Christian Ruebe

**Affiliations:** 1 Dept. of Radiooncology, Saarland University Hospital, Kirrberger Str.1, 66424 Homburg/Saar, Germany

## Abstract

**Purpose:**

To retrospectively evaluate the results after a regimen of surgery, IORT (intraoperative radiotherapy), and EBRT (external beam radiotherapy) for soft-tissue sarcomas

**Methods:**

38 consecutive patients underwent IORT for soft-tissue sarcoma; 29 were treated for primary tumours, 9 for recurrences. There were 14 cases with liposarcomas, 8 with leiomyosarcomas, 7 with malignant fibrous histiocytomas. 27/38 tumours were located in the extremities, the remaining ones in the retroperitoneum or the chest. Radical resection was attempted in all patients; a R0-resection was achieved in 15/38 patients, R1 in 12/38 pats and R2 in 4/38 pats. IORT was performed using a J-125 source and a HDR (high dose rate) afterloading machine after suturing silicone flaps to the tumour bed. The total dose applied ranged from 8–15 Gy/0.5 cm tissue depth measured from the flap surface. After wound healing external beam radiotherapy (EBRT) was applied in 31/38 patients with total doses of 23–56 Gy dependent on resection status and wound situation. The mean duration of follow-up was 2.3 years.

**Results:**

A local recurrence was found in 10/36 patients, lymph node metastases in 2/35, and distant metastases in 6/35 patients. The actuarial local control rate was 63%/5 years. The overall survival rate was 57%/5 years. There was no statistically significant difference between the results after treatment for primaries or for recurrences. Late toxicity to the skin was found in 13/31 patients, wound healing problems in 5/31 patients. A neuropathy was never seen.

**Conclusion:**

The combination of surgery, IORT, and EBRT yields favourable local control and survival data which are well within the range of the results reported in the literature. The complication rates, however, are considerable although the complications are not severe, they should be taken into account when therapy decisions are made.

## Introduction

Intraoperative radiotherapy (IORT) is known to be a reasonable therapeutic option in the treatment of soft-tissue sarcomas especially because it enables the application of higher total doses to the target volume than possible with EBRT alone, or makes possible a lower EBRT target dose with corresponding lower dose to surrounding healthy tissues. A higher local dose to the tumour bed is expected to increase the probability of local control and – at the same time – to avoid higher toxicity rates to the healthy surrounding tissues, because these can easily be removed out of the IORT target volume [1-3].

In principle, IORT can be applied using electrons of a linear accelerator situated in the operating theatre or nearby [4] or of special electron accelerators like Novac7™ [5] or Mobetron™ [6]. Another possibility is the application of brachytherapy using a Ir-192 source guided by needles or plastic tubes within silicone flaps which are very useful to maintain the irradiation geometry [7]. Lastly, some French groups prefer the implantation of plastic tubes directly into the tumour bed which allow radiotherapy by insertion of Ir-192 sources immediately or even days after surgery [8,9].

The purpose of this retrospective evaluation was to review the Homburg experience with intraoperative brachytherapy combined with EBRT for soft-tissue sarcomas and to compare our data with data taken from the literature.

## Patients and methods

### Patient characteristics

We retrospectively reviewed the data of 38 consecutive patients who underwent IORT for soft-tissue sarcoma. 29/38 patients were treated for primary sarcomas, in the remaining nine the disease had recurred. The mean age at the beginning of treatment was 56 years, the mean Karnofsky performance status 92%. In the majority of cases the sarcomas were located in the lower extremities or the retroperitoneum. The most frequent histological type was liposarcoma. The histopathological grading was predominantly G3. 30/38 tumours were classified as T2 while the subclassification in T2a or T2b was not possible because a lot of data on this point were missing in the older records.

Before definitive surgery followed by IORT, 19/28 patients with primaries had undergone inadequate surgery (14 pats.) or neoadjuvant EBRT (1 pat), or surgery and neoadjuvant radiotherapy (1 pat.). Three further pediatric patients had undergone chemotherapy before IORT: one with rhabdomyosarcoma according to the CWS protocol, two with Ewing's sarcoma according to the Euro-Ewing or Ewing-99 protocols, respectively.

The patients with recurrences had been pre-treated by surgery (3 pats) or surgery and radiotherapy (5 pats.). One pediatric patient suffering from an Ewing's sarcoma had undergone surgery, radiotherapy and chemotherapy according to the EICESS-protocol. Further details are given in Table S1, Additional file 1.

### Methods

In all patients radical resection of the tumours was attempted. The operations were performed in the departments of general surgery, trauma surgery and orthopedics of the Saarland University Hospital and resulted in a histopathologically radical resection in 15/38 pats. whereas in 12 patients the resection ended R1 and in 4 patients R2; in the remaining 7 patients this information was not available or could not be stated by the pathologist.

**Figure 1:**
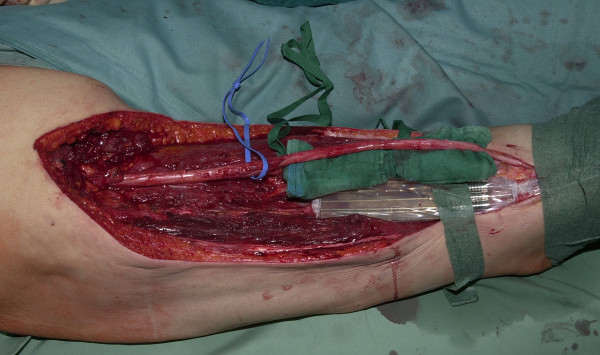
**Status after tumour resection in the lower limb, flap in position, sciatic nerve distanced**.

IORT was performed using a Gammamed12i™ high-dose-rate afterloading machine (Varian Medical Systems, Haan, Germany) with a Ir-192 source and a nominal activity of 10 Ci which was situated in a special room in direct vicinity to the operation theatre. After completion of surgery, a silicone flap (size 10 × 11 cm, in 2 patients two such flaps combined in order to cover an area of 20 × 11 cm, thickness 1 cm, containing parallel centered channels 1 cm apart and 0,5 cm from the surface) was prepared by inserting needles into the channels and connecting these to the transfer tubes. The flap was inserted into the wound and fixed to the tumour bed by sutures (see Fig. 1). Organs at risk (small bowel, large bowel, nerves) and the skin edges were kept in a safe distance. Titanium clips were fixed to the surrounding tissue near the flap corners in order to facilitate planning of EBRT later. X-rays were taken normally in anterior and lateral direction. The patient was transferred to the radiotherapy room under anaesthesia, and radiotherapy was performed there (total dose 8–15 Gy at 0.5 cm tissue depth measured from the flap surface, duration of therapy 15–48 minutes depending on dose, area to be covered, and the activity of the source on that day). In the meantime, the anesthesist monitored the patient by a camera and telemetry devices. After completion of radiotherapy, the patient was taken back to the operating theatre, x-rays were taken again in order to exclude dislocation of the flap, the flap was removed, and the wound was closed.

EBRT was intended in all patients not irradiated before, with a planned dose of 50 Gy if R0 and 56 Gy if R1 resection. Vacuum positioning devices were used regularly, mostly a 3-D therapy plan was performed based on the CT in therapy position combined with the radiographs taken in the operating theatre. For treatment we applied 6 MV X-rays of a linear accelerator.

In fact, 31/38 patients received EBRT with total doses ranging from 23–56 Gy afterwards, while the time interval between IORT and EBRT amounted to a mean of 33 (13–102) days depending on the wound healing process. The remaining 7 patients had either been irradiated neoadjuvantly (2 patients) or during therapy of the former primary tumour (5 patients).

The first follow-up examination was performed 6–8 weeks after completion of radiotherapy and then in 3 – 12 months' intervals. Regularly, a clinical examination was performed followed by ultrasound, CT or MRT. Chest X-rays were intended yearly. Force and function of the affected extremity were not recorded regularly. Overall, the data concerning toxicity were rather rare so that only late skin toxicity and delayed wound healing can be reported here.

The mean duration of follow-up was 2.3 years (0.1–10 years).

Further details are given in Table S1, Additional file 1.

All data were entered into a special medical database (MEDLOG, Parox Comp., Muenster, Germany). If follow-up data were missing written questionnaires were sent to the patients' doctors and the local authorities. Means, absolute and relative frequencies were computed. Survival curves were obtained using the Kaplan-Meier estimate and were compared using the Mantel-Haensel test. The search for prognostic factors was performed univariately using Spearman's rho and Kendall's tau tests as well as multivariately using the Cox regression hazard model.

All patients had given their written informed consent before radiotherapy. An approval by the local ethics committee was not necessary due to the retrospective evaluation. The research carried out here is in compliance with the declaration of Helsinki.

## Results

During follow-up a local recurrence was diagnosed in 10/36 patients in which sufficient data on this point could be obtained. Among the seven patients with recurrences and sufficient data, the surgical result was R2 in two, R1 in three and R0 only in 2 patients whereas among the 23 patients without a recurrence the result was R2 in two, R1 in eight and R0 in 13 patients (p = 0.0766 chi-square test).

Lymph node metastases were found in only 2/35 and distant metastases in 6/35 patients with sufficient data. In four patients, lung metastases were diagnosed, in the remaining two liver, peritoneal and lymph node metastases were found. There was no significant difference between the patients treated for a primary or for a recurrence. The actuarial local control was 63%/5 years.

**Figure 2:**
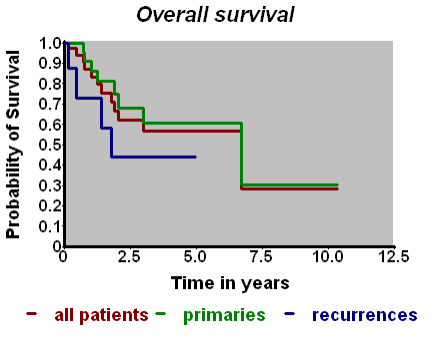
**Overall survival vs. time (Kaplan-Meier-Estimate)**.

**Figure 3:**
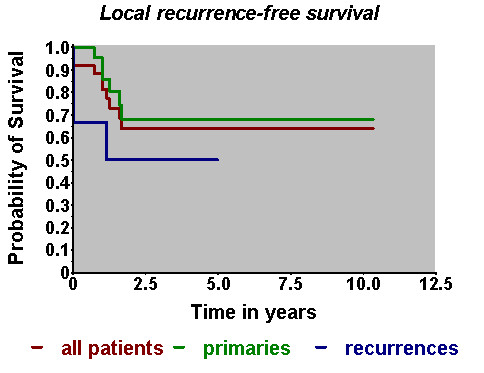
**Local recurrence-free survival (Kaplan-Meier-Estimate)**.

At the end of follow-up (mean duration: 2.3 years) 12 patients had died, 25 were known to be alive, the survival status of the remaining patient was unclear. The overall survival probability amounted to 67%/2 years and to 57%/5 years (Fig. 2). The actuarial local control rate was 64%/5 years (Fig. 3). Using the Kaplan-Meier estimate, the curve for the patients with relapses seemed to be slightly inferior to that for the patients with primaries, a statistically significant difference could not be found, which may additionally be due to the limited number of patients in the second group.

Acute gastrointestinal toxicity was rare and mild (only in 3/38 patients). Toxicity to the skin after IORT and EBRT was found as follows: no toxicity in 16/37 pats., grade I WHO in 8/33 pats., grade II WHO in two patients, and grade III WHO in 11/37 pats, whereas the higher grades of skin toxicity were found in patients with sarcomas of the extremities. Long-term side effects to the skin were found as follows: no toxicity in 18/31 pats., grade 1 EORTC in 11/31 and grade II EORTC in 2/31 patients. Patients with more intense acute side effects experienced long-term side effects more frequently and intensely (Spearman's rho, Kendall's tau, p = 0.045/0.009).

Severe wound healing problems were found in five patients with sarcomas of the extremities, one of them suffering from a small fistula in the scar, in a further three limb edema was diagnosed. Further data concerning force and function of the involved extremity are not available. We never were aware of a neuropathy.

Significant prognostic factors could be found neither univariately nor multivariately. This may be due to the small number of patients and consequently events during follow-up.

Further details are given in Table S2, Additional file 2.

## Discussion

One of the pioneers in IORT were Abe et al. working in Kyoto, Japan, who reported the method and preliminary results of application of Co-60 and electron beams directly to the tumour in the late sixties and early seventies of the last century. The authors applied IORT mainly to the stomach, the pancreas and the colon. To our knowledge, the first case report of IORT for soft-tissue sarcoma appeared in 1973 [10]. The method has been reported in more detail in 1975 [11] where the first ten cases of soft tissue sarcoma were evaluated; this collective was reanalyzed in 1980 [12]. In the meantime numerous retrospective papers were published on the subject (see Table S3, Additional file 3). The majority of author groups show that very favourable results can be obtained by a regimen of surgery, IORT, and EBRT. However, randomized trials comparing this therapy regimen to the standard (surgery followed by EBRT) are rare, trials comparing the two methods of IORT (flaps versus electron fields) are still lacking.

The majority of authors report about the intraoperative application of electrons [11-32]. While Abe et al. tried to control the tumour by IORT alone applying doses ranging from 30–45 Gy [11,12], the authors of more recent papers preferred the combination of IORT as an early boost with a highly conformal external beam radiotherapy. In this setting electron doses of 7.5 – 25 Gy were combined with doses ranging from 36 to 60 Gy applied percutaneously. The results were remarkable, the author groups show local control rates ranging from 40 to 100%/5 years resulting in overall survival data ranging from 45–84%/5 years.

Acute and long-term side effects are frequently reported. Mostly wound healing problems, gastrointestinal side effects and neuropathies are stated with the frequency of those ranging from 5 to more than 50% of the patients.

11 author groups reported about IORT using brachytherapy [8,9,22,33-40]. Typical flap techniques as described above were used by seven author groups, the remaining four applied "intraoperative implants" (2), ribbons (1) and tubes in a mesh (1). The doses applied ranged from 8 to 34 Gy in 0.5 or 1.0 cm distance from the applicator surface. EBRT doses of 0 – 50 Gy were added. The results were encouraging and within the same range as the electron results. The local control probability was found to be in the range 62–89%/5 years whereas the overall survival was 45–82%/5 years. The complication rate was considerable. Wound healing problems in 30–40% were stated, late complications in general in 24–44% of the patients.

Two author groups utilized 100 kV [17] or 250 kV [41] orthovoltage beams applying total doses of 6–25 Gy followed by an EBRT with total doses of 31–50 Gy and recorded similar results.

Our results fit well to the literature data, having achieved a local control rate of 63%/5 years and an overall survival of 57%/5 years with a late complication rate of 42% comprising delayed wound healing and late skin reactions, but neuropathy was never observed.

To our knowledge the only randomized study was conducted by Sindelar et al. [27]. The authors compared the effects of IORT + EBRT to those of EBRT alone after surgery for retroperitoneal sarcomas. They found an impressive but nevertheless insignificant gain of local control (but not of survival) after IORT + EBRT, the local complications were significantly increased after EBRT alone compared to IORT + EBRT (further details are given in Table S3, Additional file 3).

According to a patterns-of-care study conducted by Kaiser et al [42] at least 24 centres in Germany are working regularly with IORT, among these are 16 universities. 11 centers use linear accelerators, 15 perform brachytherapy. In the majority of cases IORT is prescribed for gastric, pancreatic, bile duct and rectal cancers as well as for soft-tissue and bone sarcomas. The total dose applied by IORT varies between 10 and 25 Gy.

## Conclusion

Our data and those taken from the literature show that soft-tissue sarcomas can be reasonably and successfully treated by radical surgery and a combination of brachytherapy IORT and EBRT. However, it should be born in mind that acute and late complication rates may be elevated by adding IORT to the therapy protocol whereas the evidence that this combination may be superior to surgery and EBRT alone concerning local control and survival is still limited

## Competing interests

The authors declare that they have no competing interests.

## Authors' contributions

MN was responsible for the design of the evaluation, checking the data, statistical evaluation, and writing of the manuscript. JF was responsible for the treatment of the majority of the patients and control of the documentation as well as review of the manuscript. NL was responsible for the plans and control of the procedures. CB was responsible for the evaluation of the patients' records, collection of the data, letters to the patients and the referring doctors, and the entry of the data to the databank system. CR critically evaluated and approved the manuscript. All authors have read and approved the final manuscript.

## Supplementary Material

Additional file 1 **Patient collective**. Detailed data about our patient collective Click here for file

Additional file 2 **Results**. Detailed data about the therapy results (local control, side effects) Click here for file

Additional file 3 **Summary of literature**. Detailed collection of literature data Click here for file
