# Supplementary material for: Intraoperative radiotherapy (IORT) combined with external beam radiotherapy (EBRT) for soft-tissue sarcomas – a retrospective evaluation of the Homburg experience in the years 1995–2007
Source: Radiat Oncol. 2009 Aug 26;4:32. doi: 10.1186/1748-717X-4-32 (PMC2739216; doi:10.1186/1748-717X-4-32)
Supplement: Additional file 1 — Patient collective. Detailed data about our patient collective [file 1748-717X-4-32-S1.doc]

| Item | All patients (n=38) | Primaries (n=29) | Recurrences (n=9) | Differences |
| --- | --- | --- | --- | --- |
| Mean age (years) | 55.8 [9-85] | 56.9 [10-85] | 52.1 [9-72] | n.s. |
| Mean Karnofsky performance status | 9.2 [6-10] | 9.3 [6-10] | 9.3 [8-10] | n.s. |
| Gender  male  female | 19  19 | 13  16 | 6  3 | n.s. |
| Histopathological type  Histiocytoma  Fibrosarcoma  Liposarcoma  Leiomyosarcoma  Rhabdomyosarcoma  Ewing´s sarcoma  Schwannoma  Epitheloid sarcoma | 7  1  14  8  2  3  2  1 | 6  1  10  6  2  2  1  1 | 1  0  4  2  0  1  1  0 | n.s. |
| Histopathological grading  G1  G2  G3  G4  Not known | 3  9  19  2  5 | 2  6  15  2  4 | 1  3  4  0  1 | n.s. |
| Localization  Thorax  Retroperitoneum  Pelvis  Shoulder  Hip  Upper extremity  Lower extremity | 1  10  6  2  3  1  15 | 0  7  4  2  3  1  12 | 1  3  2  0  0  0  3 | n.s. |
| Max. diameter (cm) | 11.1 [2.5-22] | 10.8 [2.5-22] | 12.2 [5-19] | n.s. |
| T-classification  (all N0/M0)  T1  T2  Not known | 6  30  2 | 5  23  1 | 1  7  1 | n.s. |
| Pre-treatment  None  Surgery  Radiotherapy  Chemotherapy  Surgery + radiotherapy  Surgery + radiotherapy +chemotherapy | 10  17  1  3  6  1 | 10  *14  **1  **3  **1  0 | 0  3  0  0  5  1 | p=0.02 |
| Surgery immediately before IORT: kind of surgery  Compartment resection  Wide excision | 33  5 | 24  5 | 0  9 | n.s. |
| Surgery immediately before IORT: R-Status  R0  R1  R2  Not known/not possible | 15  12  4  7 | 13  10  4  2 | 2  2  0  5 | n.s. |
| Dose of IORT (Gy/0.5cm tissue depth from the flap surface)  8  10  12  15 | 3  32  2  1 | 3  24  1  1 | 0  8  1  0 | n.s. |
| Mean time interval between IORT and EBRT (days) | 33 | 32 | 41 | n.s. |
| Total dose of EBRT in Gy  (single fractions of 1.8-2.0Gy, ICRU reference point)  0  23  29  45  46  50  52  56  58 | 7  1  1  5  1  13  2  7  1 | 2  0  0  4  1  12  2  7  1 | 5  1  1  1  0  1  0  0  0 | p=0.034 |

Table 1: Patient characteristics

Abbreviations:

* insufficient surgery which had to be repeated

** neoadjuvant radiotherapy/chemotherapy

IORT: intraoperative radiotherapy

EBRT: external beam radiotherapy
