# Supplementary material for: Intraoperative radiotherapy (IORT) combined with external beam radiotherapy (EBRT) for soft-tissue sarcomas – a retrospective evaluation of the Homburg experience in the years 1995–2007
Source: Radiat Oncol. 2009 Aug 26;4:32. doi: 10.1186/1748-717X-4-32 (PMC2739216; doi:10.1186/1748-717X-4-32)
Supplement: Additional file 2 — Results. Detailed data about the therapy results (local control, side effects) [file 1748-717X-4-32-S2.doc]

| Item | All patients (n=38) | Primaries (n=29) | Recurrences (n=9) | Differences |
| --- | --- | --- | --- | --- |
| Local result during follow-up  remission  relapse  not known | 26  10  2 | 22  6  1 | 4  4  1 | n.s. |
| Lymph node metastases during follow-up  absent  present  not known | 33  2  3 | 26  1  2 | 7  1  1 | n.s. |
| Distant metastases during follow-up  absent  present  not known | 29  6  3 | 23  4  2 | 6  2  1 | n.s. |
| Acute toxicity of IORT:  Nausea/Diarrhoea | 3 | 2 | 1 | n.s. |
| Acte side effects to the skin  none  1°WHO  2°WHO  3°WHO  not known | 16  8  2  11  1 | 9  8  2  10 | 7  0  0  1 | p=0.013 |
| Long-term side effects to the skin  None  1°EORTC  2°EORTC  unknown | 18  11  2  7 | 14  11  1  3 | 4  0  1  4 | n.s. |
| Limb edema | 3 | 2 | 1 | N/a |
| Wound healing problems  In total | 5/33 | 3/25 | 2/8 | N/a |

Table 2: Results in detail

Abbreviations:

EORTC: European organization for radiation therapy of cancer

N/a: not applicable: too few data for statistical analysis
