# Supplementary material for: Intraoperative radiotherapy (IORT) combined with external beam radiotherapy (EBRT) for soft-tissue sarcomas – a retrospective evaluation of the Homburg experience in the years 1995–2007
Source: Radiat Oncol. 2009 Aug 26;4:32. doi: 10.1186/1748-717X-4-32 (PMC2739216; doi:10.1186/1748-717X-4-32)
Supplement: Additional file 3 — Summary of literature. Detailed collection of literature data [file 1748-717X-4-32-S3.doc]

| **Authors**  **Study** | **Kind of surgery** | **Dose of IORT (Gy)** | **Dose of EBRT (Gy)** | **Local control** | **Overall**  **Survival** | **Complications** | **Remarks** |
| --- | --- | --- | --- | --- | --- | --- | --- |
| Abe et al. [12]  (1980)  n=15  retrospective | debulking | Electrons 30-45 | 0 | 14/15 | 4/15 died from lung metastases, 1/15 died from local recurrence | not given |  |
| Abe et al. [11] [(1975)  n=10  retrospective | debulking | Electrons  30-45Gy | 0 | 9/10 pats : 7months-2 years | not given | not given | recurrent tumours |
| Alektiar et al. [35] (2000)  n=32  retrospective | 30/32 complete gross resection | Flab  12-15Gy/1cm distance from the source | 45-50 | 62%/5 years | 45%/5 years | 11/32 mostly gastrointestinal | retroperitoneal sarcomas  2 pats. with permanent implants |
| Azinovic et al. [14]  (2003)  n=45  retrospective | marginal in13% | Electrons  10-15 | 40-60 | 88%/ 5 years (R0)  57%/5 years (R+) | 75%/ 7years | 11% neuropathy | extremity preservation rate 88%  Chemotherapy |
| Bobin et al. [31] (2003)  n=24  retrospective | complete resection in 20% | Electrons  8-22 | 45-50 | 11/22 pats./4-42 months  28%/5 years | 4-128 months  56%/5 years | neurotoxicity 6/24, severe 2/24 pats. | retroperitoneal sarcomas |
| Calvo et al. [15]  (1995)  n=30  retrospective | Maximal resection  9/30 R2 | Electrons  10-20 | 0-50 | R0 : 65%  R>0: 35% | primaries : 53%/5years  recurrences : 20%/5 years | 1 myelopathy  3 x neuropathy  2 fistulae | central sites  Chemotherapy in ca. 50% of the patients |
| Delannes et al. (2000) [8]  n=58  retrospective | 37/58 wide margins | intraoperative implant  12-25 | 45-50 | 89%/5 years | 65%/5 years | wound healing problems in 20/58 pats, late side effects in 16/58 pats (7 neuropathies) | Chemotherapy in 28/58 pats. |
| Dibiase et al. [36] (1997)  n=14  retrospective | debulking | Brachy  Ir-192 ribbons  14-35 | 0-70 | 11/13 pats /6months | not given | acute dermatitis in 1/14 pats. | diverse tumours  vicryl mesh |
| Dubois et al. [17] (1995)  n=31  retrospective | 30/31 complete resection | 100 kVx, electrons, 10-25 | 45-50 | 87% | 65%/ 5years | 1/31 : rupture of the femoral artery after 25Gy |  |
| Dziewirski et al. [37] (2006)  N=46  retrospective | Wide excision  65% R0 | HDR implant (flab)  20/1.0cm | 36-50 | 51%/5 years | 51%/5 years | Complications requiring surgery in 21.7% | Retroperitoneal sarcomas |
| Gieschen et al. [18] (2001)  n=37  retrospective | 78% gross total resection | Electrons  10-20 in 20/37 patients | 45-50 | 59%/5 years | 50%/ 5 years | 4/20 significant late complication (neuropathy, fistula, small bowel obstruction, ischemia | neoadjuvant radiotherapy followed by surgery and IORT, clear benefit for patients with IORT |
| Gilbeau et al. [19]  (2002)  n=45  retrospective | 38% R0 | Electrons  13-30 | 40-59.4 | 40%/5 years | 60%/ 5years | 30-42% | IORT vs. no IORT  Chemotherapy in11 patients. Survival and local control data for the whole collective. No benefit for IORT |
| Gunderson et al. (1982) [32]  n=36  retropective | debulking | Electrons  10-17.5 | 45-50 | 17/21 | 11-30 months | 16/36 | diverse tumours |
| Haddock et al. [20] (1997)  n=91  retrospective | total resection | Electrons 7.5-20 | 19.8-59.4 | 92%/3years | 76%/3years | not given | chemotherapy in 24/91 pats. |
| Houtmeyers et al. (2007) [34]  retrospective | debulking | Flab  8/1cm | 0 | 2-44 months | 3-48 months | acute 4/10 pats  late : none | diverse tumours  recurrent or unresectable intraabdominal malignancies  all patients had undergone radiotherapy before |
| Jones et al. (2002) [33]  N=55  Prospective | Tumor and contiguous structures | PDR implant (tubes in a mesh)  7.3-30/0.5cm | 42-50 | Disease-free survival 79.5%/2 years | 73%/2 years | Gastrointestinal toxicity >=III°: 4/42 pats. | Retroperitoneal sarcomas:  neoadjuvant EBRT followed by surgery and brachytherapy |
| Könemann et al. [39] (2002)  n=31  retrospective | not given | TEBA (tissue equivalent bendy applicator  0-24Gy/0-8 fractions | 32-60/17 pats | not given | not given | seroma, hematoma, neuropathy in 4 pats | focus on desciption of method |
| Krempien et al. [21] (2006)  N=67  Retrospective | Wide excision,  31% R0 | Electrons  12-20 | 20-59.4 | 40%/5 years | 64%/5 years | Late toxicity grades II-IV in 21%, 5 patients with neuropathy | Retroperitoneal sarcomas |
| Kretzler et al. [22] (2003)  n=28  retrospective | R0 in 61% | Flab (n=16)  Electrons  (n=12)  12-15 | 30.6-60 | 85%/ 5years | 66%/ 5years | 18% wound healing problems, 24% grade 3-4 late effects (neuropathy, fractures) |  |
| Kunos et al. [23] (2006)  n=27  retrospective | marginal excision (15%)  wide local excision (85%) | 10-12  Electrons | 50-63 | not given | not given | within 90 days: wound dehiscence 14 vs.15%  total complicatons 36 vs.15% (n.s.)  Late complications 21 vs. 39% (n.s.) | preop. vs postop. EBRT |
| Lehnert et al. [24] (2000)  n=92  retrospective  extremities  retroperitoneum  trunk | 74% R0  31% R0  69% R0 | Electrons  12-18 | 40 | 83%/5 years  60%/5 years  100%/5 years | 78%/5 years  52%/5 years  50%/5 years | 23% | IORT vs. no IORT : clear benefit for local control, not for overall survival ; complications enhanced |
| Llácer et al. [9] (2006)  n=79  retrospective | 31/79 R0 | intraoperative implant  LDR  10-45Gy  start median 4 days after surgery | 45-50Gy | 90%/5 years | 69%/5 years | acute : 30%  late : 44% | Chemotherapy in 38/79 pats. |
| Oertel et al. [25] (2006)  N=153  retrospective | Wide excision, 49% R0 | Electrons  10-20 | 36-50.4 | 78%/5 years | 77%/5 years | Late toxicity grades II-IV in 17%, 5% neuropathy | Extremity sarcomas |
| Pierie et al. [13] (2006)  n=103  retrospective | complete resection in 62/103 pats | Electrons 10-20 | 40-50 | 24/62 pats. after complete resection | 48%/5years  62% (complete resection)  29%(debulking) | 5/103 pats. | retroperitoneal sarcomas  chemotherapy in 24/103 pats. |
| Rachbauer et al. [40] (2002)  n=39  retrospective | all marginal | Flab  10/0.5cm | 50 | disease-free  84%/ 2years  no local relapses | 82%/ 2years | no IORT-related  wound complications in 28% | chemotherapy in 21 pats. |
| Richter et al. [26] (2003)  n=78 (with IORT n=26)  retrospective | not given | Electrons 20Gy | not given | IORT 15%, no IORT 29% | 3.8 years/IORT  5.75 years/ no IORT | late: 34%/IORT7%/no IORT | chemotherapy in 28 pats. |
| Schuck et al. (1997) [38]  n=20  retrospective | total resection | Flab  10/0.5cm | 0 | 19/20  distant metastases in 7/20 pats. | not given | 4/20 : delayed wound healing | neoadjuvant radiochemotherapy  45-55Gy |
| Sindelar et al. [27] (1993)  n=35  randomized | marginal resection | Electrons  20  0 | 35-40  50-55 | median time  63 months  38 months  n.s. | median time  45 months  52 months  n.s. | gastrointestinal  1/15 pats  12/20 pats  p<0.01 | retroperitoneal sarcoma  IORT + EBRT vs.  EBRT alone |
| Tran et al. (2006) [28]  n=17  restrospective | 11/16 negative margins | Electrons  12-15 | 13/17 applied  43.2-61.2 | 86%/3 years | 78%/ 3 years | no acute toxicity |  |
| Tran et al. [41] (2008)  N= 50  Retrospective | Wide excision, 70% R1, 15% R2 | 200-250kV Orthovoltage beam  6-16/Surface | 31-50 (37%) | Not given | 55%/5 years | 10% with grade III/IV late toxicity | 78% retroperitoneal sarcomas |
| van Kampen et al. [29] (2001)  n=53  retrospective | all wide excision | Electrons 12.5-20 | 40-50 | 90%/ 5 years | 84%/5 years | 11/53 soft tissue fibrosis | toxicity depending on volume irradiated |
| Willet et al [30] (1991)  n=20  retrospective | 14/20 compete resection | Electrons  10-20  in12/20 pats. | 40-50 | 81%/ 4 years | not given | 5/20 pats. |  |
|  |  |  |  |  |  |  |  |

Abbreviations:

EBRT = external beam radiotherapy

PDR = pulsed dose rate

HDR = high dose rate
